# Global Pharmacovigilance of Aesthetic Botulinum Toxin Type A: Analysis of Adverse Event Reports From the USA, Europe, Canada, and Australia

**DOI:** 10.1111/jocd.70771

**Published:** 2026-04-12

**Authors:** Alexander Zargaran, David Zargaran, Ali Pirayesh, Alex Woollard, Afshin Mosahebi

**Affiliations:** ^1^ University College London London UK; ^2^ Royal Free Hospital London UK

**Keywords:** adverse events, botulinum toxin, complications, global health, pharmacovigilance

## Abstract

**Background:**

Monitoring of adverse events for aesthetic BoNT‐A (BoNT‐A) through pharmacovigilance databases provides critical insight into real‐world safety profiles. Previous analyses suggested substantial underreporting, yet comprehensive international multi‐database analysis remains absent.

**Methods:**

Adverse event reports for aesthetic BoNT‐A indications were extracted from FDA FAERS, EMA EudraVigilance, Health Canada MedEffect, and TGA DAEN databases from 2002 to 2025. Reports were filtered for cosmetic indications using procedure codes, anatomical sites, and indication descriptions. Events were categorized by clinical manifestation and severity using MedDRA classification.

**Results:**

Analysis identified 43,809 adverse event reports across four databases. FDA FAERS contributed 38,321 reports (87.5%), EudraVigilance 3,526 (8.0%), Health Canada 1,835 (4.2%), and TGA DAEN 127 (0.3%). Effectiveness issues comprised 57.3% of reports (*n* = 25,092), followed by local injection‐site reactions (19.7%, *n* = 8,611), facial paresis/ptosis/asymmetry (9.9%, *n* = 4,348), and headache (6.0%, *n* = 2,650). Peak reporting occurred in 2018 for FAERS (1,375 reports) and 2020 for Health Canada (242 reports). Among FAERS reports with brand attribution, onabotulinumtoxinA accounted for 86.8%, abobotulinumtoxinA 9.7%, and incobotulinumtoxinA 2.4%. Co‐reported adverse event analysis revealed drug ineffective with therapeutic response decreased as the most frequent pairing (*n* = 2,947), followed by drug ineffective with off‐label use (*n* = 1,102).

**Conclusions:**

The predominance of effectiveness issues over traditional safety concerns distinguishes aesthetic BoNT‐A pharmacovigilance from typical pharmaceutical surveillance. Geographic concentration of reporting in the United States despite global market distribution indicates systematic underreporting internationally. These findings suggest current pharmacovigilance systems inadequately capture aesthetic medicine adverse events, with direct implications for patient safety and regulatory oversight.

## 1 Introduction

Monitoring of adverse events for aesthetic BoNT‐A type A (BoNT‐A) through pharmacovigilance databases provides essential real‐world safety data complementing controlled clinical trials. Estimates of demand for aesthetic BoNT‐A vary, with the American Society of Plastic Surgeons (ASPS) quoting over 9.8 million procedures in the USA alone in 2024, a 4% increase from the previous year [1], whilst ISAPS (The International Society of Aesthetic Plastic Surgery) global 2024 survey quoted over 7.8 million procedures worldwide in 2023 [2]. This reflects the fragmented nature of practice and governance, both within countries and internationally. Both sources agree that aesthetic BoNT‐A is the most performed aesthetic procedure worldwide, with ASPS citing its affordability as a major driving force behind this.

There is international heterogeneity in regulations and enforcement across jurisdictions [3]. The public health implications of that variation are already visible in cross‐border events. For example, the United Kingdom's Medicines and Healthcare products Regulatory Agency seized more than 4700 counterfeit BoNT‐A vials between 2023 and 2025, traced to unlicensed imports from Asia and linked to over 40 hospitalisations [4]. This demonstrates how weaknesses in one jurisdiction can have international consequences in a globally interconnected market.

Previous analyses of individual pharmacovigilance databases have revealed concerning patterns of underreporting. The UK Medicines and Healthcare products Regulatory Agency received only 188 adverse event reports over a 20‐year period, contrasting with a meta‐analysis demonstrating complication rates of approximately 16% across studies [5], with a further study exploring the lived experience of those suffering from complications identifying that complications range from mild and transient to severe and debilitating, encompassing physical, financial, and psychological [6]. This discrepancy between spontaneous reporting rates and documented adverse event incidences raises fundamental questions about pharmacovigilance adequacy for cosmetic interventions occurring predominantly outside traditional healthcare settings.

The regulatory classification of BoNT‐A as a prescription‐only medication creates requirements for adverse event reporting comparable to other pharmaceuticals. However, the commercial context of aesthetic medicine, where procedures are performed for cosmetic enhancement rather than medical necessity, may alter reporting behaviors among practitioners and patients. Unregulated practitioners operating in competitive aesthetic markets may perceive adverse event reporting as threatening practice reputation, while patients may not recognize mild complications as reportable events.

This study presents a comprehensive analysis of 43,809 adverse event reports related to aesthetic BoNT‐A administration extracted from four major international pharmacovigilance databases between 2002 and 2025. The analysis examines distribution patterns, clinical manifestations, and temporal trends to provide the most complete assessment of aesthetic BoNT‐A safety data available from spontaneous reporting systems.

## 2 Methods

2.1

Adverse event data were systematically extracted from four international pharmacovigilance databases. The United States Food and Drug Administration (FDA) Adverse Event Reporting System (FAERS) [7] provided data from 2002 to 2024. The European Medicines Agency (EMA) EudraVigilance [8] system contributed reports from 2004 to September 2025. Health Canada MedEffect [9] database provided reports from 2002 to 2024. The Australian Therapeutic Goods Administration (TGA) Database of Adverse Event Notifications (DAEN) [10] supplied data from 2002 to 2024.

2.2

Reports were included if they specified aesthetic or cosmetic indications for BoNT‐A products. Filtering employed procedure codes indicating cosmetic use, anatomical injection sites consistent with aesthetic applications (glabellar lines, forehead lines, lateral canthal lines), and explicit cosmetic indication descriptions. Reports for therapeutic indications including chronic migraine, hyperhidrosis, dystonia, and neurogenic bladder were excluded. Unclear cases where injection patterns suggested aesthetic use were retained to minimize exclusion of relevant reports.

2.3

Extracted variables included report date, adverse event terms, product identification, and reporter type where available. Adverse events were classified using the Medical Dictionary for Regulatory Activities (MedDRA) preferred terms and grouped into clinical categories. Primary categories comprised effectiveness issues (drug ineffective, therapeutic response decreased), local injection‐site reactions, neuromuscular effects (ptosis, facial asymmetry), and systemic manifestations.

2.4

Descriptive statistics characterized adverse event distributions across databases, time periods, and clinical categories. Temporal trends were assessed through annual reporting frequencies. Co‐reported adverse events were analyzed to identify patterns of associated complications. Brand‐specific analyses compared adverse event profiles between different BoNT‐A formulations where product attribution was documented.

## 3 Results

3.1

**FIGURE 1:**
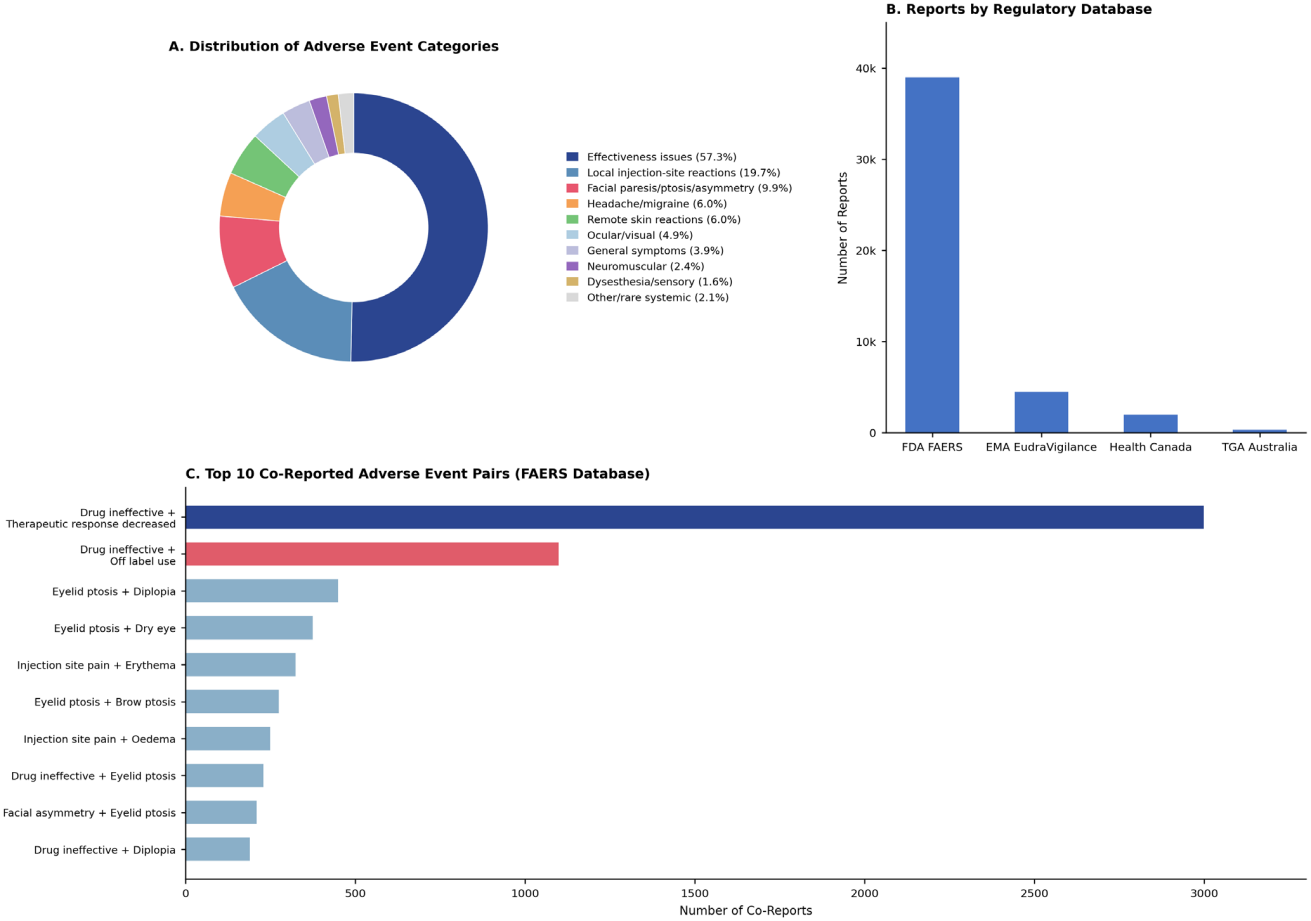
Distribution of 43,809 adverse event reports for aesthetic BoNT‐A type A from international pharmacovigilance databases (2002–2025). (A) Distribution of Adverse Event Categories. (B) Reports by Regulatory Database. (C) Top 10 co‐reported adverse event pairs from the FAERS Database.

Analysis of combined pharmacovigilance databases identified 43,809 adverse event reports associated with aesthetic BoNT‐A between 2002 and September 2025 (Table 1). The FDA FAERS database contributed the vast majority with 38,321 reports (87.5%), followed by EMA EudraVigilance with 3,526 reports (8.0%), Health Canada MedEffect with 1,835 reports (4.2%), and TGA DAEN with 127 reports (0.3%). Figure 1 presents the comprehensive distribution of adverse events across categories and databases.

**TABLE 1:** Cosmetic BoNT‐A adverse event reports by database (2002–2025).

Database	Years covered	Total reports	Peak year	Peak count	% of total
FDA FAERS	2002–2024	38,321	2018	1,375	87.5%
EMA EudraVigilance	2004–2025^a^	3,526	2025	637	8.0%
Health Canada	2002–2023	1,835	2020	242	4.2%
TGA Australia	2002–2023	127	2018	10	0.3%
Total	2002–2025	43,809	—	—	100%

^a^ 2025 data partial through September.

Temporal analysis revealed distinct reporting patterns across databases. FDA FAERS demonstrated progressive increases from fewer than 500 annual reports in the early 2000s to peak reporting of 1,375 in 2018, followed by moderate decline to approximately 900 reports annually by 2023. Health Canada showed gradual growth from 15 reports in 2002 to 242 in 2020. EMA EudraVigilance exhibited minimal reporting before 2010, with subsequent increases reaching 637 reports in the first 9 months of 2025. Australian TGA maintained consistently low reporting throughout the study period.

3.2

The combined analysis revealed a distinctive adverse event profile dominated by effectiveness concerns rather than traditional safety issues (Table 2). Effectiveness issues, comprising “drug ineffective” and “therapeutic response decreased”, constituted 57.3% of all reports (*n* = 25,092). Local injection‐site reactions including pain, erythema, swelling, bruising, and haematoma represented 19.7% (*n* = 8,611). Neuromuscular effects manifesting as facial paresis, ptosis, and asymmetry accounted for 9.9% (*n* = 4,348).

**TABLE 2:** Distribution of adverse event categories (*N* = 43,809).

Adverse event category	Number of cases	% of reports
Effectiveness issues	25,092	57.3%
Local injection‐site reactions	8,611	19.7%
Facial paresis/ptosis/asymmetry	4,348	9.9%
Headache/migraine	2,650	6.0%
Remote skin reactions	2,620	6.0%
Ocular/visual disturbances	2,156	4.9%
General symptoms	1,689	3.9%
Neuromuscular symptoms	1,040	2.4%
Dysesthesia/sensory	693	1.6%
Other/rare systemic events	≤ 300 each	< 1%

Headache and migraine were reported in 6.0% of cases (*n* = 2,650). Remote skin reactions including rash, urticaria, and oedema occurred in 6.0% (*n* = 2,620). Ocular and visual disturbances comprising diplopia, dry eye, epiphora, lagophthalmos, and vision disturbance affected 4.9% (*n* = 2,156). General symptoms including fatigue, asthenia, fever, and flu‐like symptoms were documented in 3.9% (*n* = 1,689). Serious systemic events including cardiovascular, respiratory, and allergic reactions each occurred in fewer than 300 cases, representing less than 1% of total reports. Categories are not mutually exclusive; a single report can include multiple MedDRA terms, so percentages exceed 100% in total.

3.3

Product attribution was available for a subset of FAERS reports, enabling brand‐specific analysis (Table 3). Among FAERS cases with brand attribution, onabotulinumtoxinA accounted for 86.8%, abobotulinumtoxinA 9.7%, incobotulinumtoxinA 2.4%, and other/unspecified 1.1%.

**TABLE 3:** Brand attribution in FAERS cosmetic dataset (2002–2023).

Brand	% of FAERS cases
OnabotulinumtoxinA (Botox)	86.8%
AbobotulinumtoxinA (Dysport)	9.7%
IncobotulinumtoxinA (Xeomin)	2.4%
Other/unspecified	1.1%

3.4

Analysis of simultaneously reported adverse events within individual cases revealed patterns of associated complications (Table 4). The most frequent co‐occurrence involved “drug ineffective” with “therapeutic response decreased”, appearing together in 2,947 cases. This pairing likely represents redundant reporting of treatment failure or patient emphasis on lack of expected results. Drug ineffective co‐occurred with off‐label use in 1,102 cases, suggesting potential association between unapproved applications and suboptimal outcomes.

**TABLE 4:** Most frequent co‐reported adverse event pairs in FAERS dataset.

Adverse event 1	Adverse event 2	Co‐reports
Drug ineffective	Therapeutic response decreased	2,947
Drug ineffective	Off label use	1,102
Eyelid ptosis	Diplopia	451
Eyelid ptosis	Dry eye	317
Injection site pain	Erythema	280
Eyelid ptosis	Brow ptosis	265
Injection site pain	Oedema	233
Drug ineffective	Eyelid ptosis	210
Facial asymmetry	Eyelid ptosis	188
Drug ineffective	Diplopia	176
Diplopia	Dry eye	162
Injection site pain	Bruising/hematoma	158
Brow ptosis	Diplopia	150
Rash	Urticaria	132
Drug ineffective	Facial asymmetry	125

Among neuromuscular complications, eyelid ptosis frequently co‐occurred with other periocular effects including diplopia (451 cases), dry eye (317 cases), and brow ptosis (265 cases). These associations reflect anatomically predictable patterns of toxin diffusion affecting adjacent muscle groups. Local injection reactions demonstrated expected clustering, with injection site pain co‐reported with erythema (280 cases), oedema (233 cases), and bruising/hematoma (158 cases).

3.5

Analysis of the top 25 co‐reported adverse event pairs revealed further patterns. Erythema with oedema occurred in 119 cases, while diplopia with vision blurred was reported in 110 cases. Injection site swelling co‐occurred with pain in 105 cases. Diplopia paired with eyelid lag in 97 cases, while muscle weakness accompanied eyelid ptosis in 91 cases. Dry eye with lagophthalmos appeared together in 88 cases, drug ineffective with headache in 83 cases, and facial asymmetry with brow ptosis in 81 cases. Drug ineffective co‐occurred with injection site pain in 77 cases, and diplopia with ptosis in 73 cases.

## 4 Discussion

A total of 43,809 adverse event reports over 23 years across four global pharmacovigilance databases represents the largest analysis of aesthetic BoNT‐A post‐marketing surveillance complications to date. Previous analysis of the UK MHRA database identified only 188 adverse events over 20 years, citing significant underreporting in view of the documented 16% complication rate [5]. The present study reinforces this finding across the USA, Europe, Australia, and Canada, indicating that international pharmacovigilance databases are likely inadequately capturing complications, with profound resulting patient safety implications.

The finding that effectiveness issues constitute 57.3% of all adverse event reports is significant. Previous studies have explored the potential immunogenicity in aesthetic BoNT‐A [11, 12], with a meta‐analysis of 43 studies citing a 1.8% incidence of Neutralizing Antibodies following BoNT‐A injection [11]. However, it is not clear from the database or reports whether the reported effectiveness issues are related to immunogenicity, inappropriate drug reconstitution, inadequate technique, or patient expectations. The association between drug ineffective and off‐label use (1,102 cases) raises important questions about outcomes when BoNT‐A is administered beyond approved indications. These patterns indicate that current pharmacovigilance systems, designed for medical adverse events, may inadequately capture the full spectrum of concerns relevant to aesthetic medicine.

The extent of underreporting is apparent when comparing pharmacovigilance records to expected complication rates derived from procedural volumes. In the United States, the American Society of Plastic Surgeons estimated 9.8 million aesthetic BoNT‐A procedures in 2024. A meta‐analysis of clinical studies has consistently found pooled complication rates of approximately 16% [5]. On that basis, more than 1.5 million adverse events would be expected in the US in 2024 alone. FAERS recorded fewer than 1,000 cosmetic BoNT‐A reports in that year, representing less than 0.1% of the estimated total.

Comparable discrepancies appear internationally. Canada reported 242 adverse events in 2020 through MedEffect, despite conducting an estimated hundreds of thousands of annual procedures. Australia's DAEN database recorded only 127 adverse events across the entire 2002 to 2023 period. EudraVigilance produced a modest number of reports relative to the size of the European market, which accounts for millions of procedures annually.

These comparisons indicate that current pharmacovigilance systems substantially under‐record adverse events and provide limited value for estimating true risk or guiding public health surveillance in aesthetic medicine.

4.1

The concentration of adverse event reporting in the United States, contributing 87.5% of global reports, cannot be explained by population alone, since the EMA EudraVigilance database received reports from countries across the European Union, which cumulatively exceeds the population of the USA. In terms of market share, the USA represents approximately 40% of worldwide aesthetic BoNT‐A procedures according to industry estimates [2], suggesting a greater than two‐fold overrepresentation in spontaneous reporting. This geographic disparity likely reflects multiple factors including regulatory environment, reporting infrastructure, cultural attitudes toward adverse event documentation, and medicolegal considerations.

The minimal contribution from the Australian TGA (0.3% of reports) despite that country's substantial aesthetic medicine market also indicates significant underreporting. Similarly, the growing contribution from EudraVigilance reaching 637 reports in 2025 suggests improving but still inadequate European reporting. These patterns demonstrate that global pharmacovigilance for aesthetic BoNT‐A remains fragmented, with vast differences in reporting culture preventing comprehensive safety surveillance.

4.2

A previous meta‐analysis identified the most common complication of facial aesthetic BoNT‐A injections to be headache and migraine, accounting for 6.3% of complications [5]. In the previous MHRA database analysis, General Symptoms of influenza‐like symptoms, asthenia, chills, pyrexia, and fatigue, accounted for the highest proportion of complications (28.2%) [13]. The most commonly reported clinical complication in the present study was local skin reactions (19.7%), whilst these only accounted for 3.8% of complications in the meta‐analysis. The higher‐than‐expected incidence of skin reactions compared with the meta‐analysis may be due to the nature of the meta‐analysis only including randomized controlled trials with specialist administration, whilst the pharmacovigilance databases include practitioners from different backgrounds who may not have the same level of training as specialists. The frequent co‐occurrence of ptosis with diplopia (451 cases), dry eye (317 cases), and brow ptosis (265 cases) demonstrates predictable patterns of periocular muscle involvement that could inform injection technique refinement.

The anatomical clustering of neuromuscular complications provides insight into diffusion patterns and high‐risk injection zones. The co‐reporting patterns suggest that when ptosis occurs, multiple adjacent muscle groups are often affected, potentially reflecting excessive dose, improper injection depth, or patient‐specific anatomical variations affecting toxin spread. These findings emphasize the importance of conservative dosing and precise injection technique, particularly in the periocular region where multiple critical structures lie in proximity.

4.3

The analysis reveals fundamental limitations of spontaneous reporting systems for aesthetic medicine surveillance. The voluntary nature of reporting, combined with the absence of denominator data on total procedures performed, prevents calculation of true incidence rates. The predominance of consumer‐accessible databases in the United States may partially explain geographic disparities, as other regions may lack similar reporting infrastructure or awareness.

The high proportion of effectiveness issues suggests that patients and practitioners may use pharmacovigilance systems differently for aesthetic treatments than for medical therapies. Patients dissatisfied with cosmetic outcomes may be more motivated to report than those experiencing mild, expected complications. Conversely, practitioners may underreport common complications they consider inherent to the procedure. These behavioral differences compromise the ability of current systems to accurately characterize the safety profile of aesthetic interventions.

4.4

These findings have important implications for enhancing pharmacovigilance in aesthetic medicine. The geographic disparities in reporting indicate need for international harmonization of reporting requirements and infrastructure development in underreporting regions. The predominance of effectiveness issues suggests that aesthetic‐specific reporting frameworks capturing both safety and efficacy outcomes could better serve surveillance needs.

## 5 Conclusions

Analysis of 43,809 adverse event reports from four international pharmacovigilance databases provides the most comprehensive analysis of aesthetic BoNT‐A safety data from national reporting databases. The predominance of effectiveness issues over traditional safety concerns distinguishes aesthetic medicine pharmacovigilance from conventional pharmaceutical surveillance. The high geographic concentration of reporting in the United States, contributing nearly 90% of global reports despite representing less than half of procedures, indicates systematic international underreporting of adverse events.

The co‐reporting patterns reveal predictable anatomical clustering of neuromuscular complications and associations between off‐label use and treatment failure that can inform clinical practice improvement. The relatively low frequency of serious systemic events, occurring in fewer than 300 cases among 43,809 reports spanning 23 years, supports the generally favorable safety profile of aesthetic BoNT‐A when administered according to approved indications.

These findings demonstrate that current pharmacovigilance systems, designed for medical adverse event surveillance, inadequately capture the full spectrum of concerns relevant to aesthetic medicine. Enhancement through aesthetic‐specific reporting frameworks, improved international harmonization, and better integration of consumer and practitioner reporting channels could address current limitations. As the global aesthetic BoNT‐A market continues expanding, particularly in currently underreporting regions, strengthened pharmacovigilance becomes essential for maintaining comprehensive safety surveillance and ensuring optimal patient outcomes.

## Author Contributions

All authors have seen and approved the manuscript, and have contributed in line with ICMJE criteria for authorship.

## Funding

The authors have nothing to report.

## Conflicts of Interest

The authors declare no conflicts of interest.

## Data Availability

The data that support the findings of this study are available in FDA FAERS at https://www.fda.gov/drugs/fdas‐adverse‐event‐reporting‐system‐faers/fda‐adverse‐event‐reporting‐system‐faers‐public‐dashboard. These data were derived from the following resources available in the public domain: FDA FAERS, https://www.fda.gov/drugs/fdas‐adverse‐event‐reporting‐system‐faers/fda‐adverse‐event‐reporting‐system‐faers‐public‐dashboard—TGA DAEN, https://www.tga.gov.au/safety‐and‐shortages/database‐adverse‐event‐notifications‐daen—MedEffects, https://www.canada.ca/en/health‐canada/services/drugs‐health‐products/medeffect‐canada/adverse‐reaction‐database/medeffect‐canada‐caveat‐privacy‐statement‐interpretation‐data‐search‐canada‐vigilance‐adverse‐reaction‐online‐database.html—EudraVigilance, https://www.ema.europa.eu/en/human‐regulatory‐overview/research‐development/pharmacovigilance‐research‐development/eudravigilance.
